# DNA methylation profiling of human CD4 ^+^ T helper cells reveals the epigenetic control of SLAMF7 expression in IFN‐γ producing cells

**DOI:** 10.1111/imcb.70063

**Published:** 2025-11-04

**Authors:** Anna Ntalli, Michael Beckstette, Saumya Kumar, Laura Maggi, Francesco Annunziato, Luis Graca, Stefan Floess, Jochen Huehn

**Affiliations:** ^1^ Department of Experimental Immunology Helmholtz Centre for Infection Research 38124 Braunschweig Germany; ^2^ Bielefeld Institute for Bioinformatics Infrastructure, Department of Technology Bielefeld University Bielefeld Germany; ^3^ Instituto de Medicina Molecular, Faculdade de Medicina Universidade de Lisboa 1649‐028 Lisbon Portugal; ^4^ Department of Computational Biology of Individualised Medicine Centre for Individualised Infection Medicine (CiiM), A Joint Venture Between the Hannover Medical School and the Helmholtz Centre for Infection Research Hannover Germany; ^5^ Department of Experimental and Clinical Medicine University of Florence 50134 Florence Italy

**Keywords:** Epigenetic regulation, methylome, T helper cells

## Abstract

Naive CD4^+^ T cells are highly plastic cells that can differentiate into various T helper (Th) cell subsets upon activation. It is well accepted that the vital expression of specific transcription factors and effector cytokines that characterize the different Th cell fates can be stabilized by epigenetic mechanisms including DNA methylation. However, a global view on DNA methylation profiles in Th cell subsets is currently lacking. In this study, we identified the DNA methylomes of human naive T cells, Th1, nonclassic Th1, and Th17 cells by performing a whole‐genome bisulfite sequencing analysis. Differentially methylated regions (DMRs) obtained by pairwise comparison of the Th cell methylomes indicate a close relationship between ncTh1 and Th17 cells on a genome‐wide level. However, similar methylation patterns at key gene loci such as *TBX21*, *IFNG*, *SLAMF7*, and *SLAMF8* may explain the functional proximity of ncTh1 to Th1 cells. Using luciferase reporter assays, we demonstrated that DNA methylation can modulate the transcriptional activity of promoter‐located DMRs belonging to genes such as *GSPT1*, *SRSF7*, *SLAMF7*, *SLAMF8*, *TIGIT*, and *PDCD1*. Upon stimulation, *SLAMF7* gene expression was upregulated exclusively in IFN‐γ producing cells, with SLAMF7^+^ cells appearing among both Th1 and ncTh1 cells. Taken together, the DNA methylomes of proinflammatory human Th cells provide useful data for better functional characterization of the lineages and identification of key genes for therapeutic intervention.

## Graphical abstract

In this study, we confirmed the close relationship between Th17 and ncTh1 cells by conducting a genome‐wide DNA methylation analysis with CD4^+^ T helper subsets. However, partial epigenetic changes enable ncTh1 cells to execute Th1‐specific transcriptional programs and express proteins such as SLAMF7. Therefore, we hypothesize that epigenetic adaptation occurs in highly inflammatory environments.

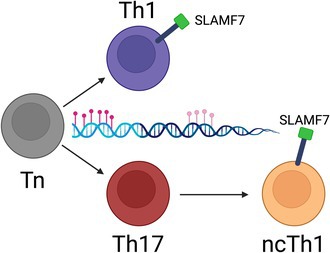

## INTRODUCTION

CD4^+^ T‐cell development from a naive state into an effector lineage requires active signal transduction pathways, which are triggered by the T‐cell receptor (TCR), costimulatory molecules, and cytokine receptors.^1^ T helper 1 (Th1) and T helper 2 (Th2) cells were the first described subsets of differentiated effector cells producing the cytokines IFN‐γ or IL‐4, respectively.^2^ A plethora of subsequent studies identified further Th cell subsets such as T helper 17 (Th17), T follicular helper, or peripheral regulatory T cells, each characterized by the expression of lineage‐specific transcription factors, cytokines, and surface molecules.^3^ Seminal studies showed an upregulation of the transcription factor T‐bet, leading to the development of IFN‐γ producing Th1 cells, whereas the expression of GATA3 initiates the Th2 cell program resulting in IL‐4 expression.^4^, ^5^, ^6^ Repetitive, instructive stimulation *in vivo* is necessary to develop naive T (Tn) cells into stable Th1 or Th2 cells, which could not be converted by exogenous factors.^7^ However, the Th1 and Th2 cell programs are not mutually exclusive as shown by the existence of T‐bet and GATA3 double‐positive cells.^8^, ^9^ In addition, more flexibility was observed in the Th cell compartment after the discovery of Th17 cells, characterized by the expression of the transcription factor RORγt and the cytokine IL‐17A.^10^, ^11^ Th17 cells can also express transcription factors and effector cytokines that are usually found in other Th cell lineages.^12^ One major pathway of Th17 cell plasticity is the switch toward IFN‐γ‐expressing cells. Restimulation of Th17 cells *in vitro* in the presence of IL‐12 or IL‐23 changes the phenotype dependent on STAT4 and T‐bet.^13^, ^14^ In murine disease models for colitis and multiple sclerosis, Th17 cells downregulate IL‐17A and upregulate IFN‐γ expression.^15^, ^16^ In humans, we previously identified nonclassic Th1 (ncTh1) cells (CD4^+^CD161^+^CCR6^+^CXCR3^+^) that originate from Th17 cells (CD4^+^CD161^+^CCR6^+^CXCR3^−^) in a TNFα‐dependent mechanism.^17^ A candidate DNA methylation approach discovered the demethylation of the conserved noncoding sequence (CNS) 1 in *TBX21* and *RORC* promoter in ncTh1 cells,^18^ located in the lineage transcription factor genes for T‐bet and RORγt, respectively. In addition, the effector cytokine loci for Th1 and Th17 cells were also affected. We have detected a demethylation at the promoter and CNS‐1 in the *IFNG* locus, whereas the *IL17A* and *IL17F* promoter were only partially demethylated. The absence of IL‐17A secretion in ncTh1 cells is presumably regulated by the transcription factor EOMES.^19^

Several studies have described the influence of DNA methylation, as part of epigenetic mechanisms, on the development and function of CD4^+^ Th cell lineages.^20^ Differentially methylated regions (DMRs) were first identified in lineage transcription factor and effector cytokine loci of Th1 and Th2 cells.^21^, ^22^ Recently, genome‐wide DNA methylation data on human *ex vivo* isolated CD4^+^ T‐cell subsets were generated, discriminating between naive and certain stages of memory cells.^23^, ^24^ However, DNA methylation profiling studies on specific human Th cell lineages are currently missing. To gain a better understanding of how proinflammatory Th cells develop into different lineages, including lineage switching, we performed genome‐wide bisulfite sequencing on naive T cells, Th1, Th17, and ncTh1 cells. Pairwise comparisons between naive and differentiated T cells revealed a higher number of DMRs in Th17 cells than in Th1 cells, indicating more epigenetic changes during Th17 cell development. Although ncTh1 cells express the cytokine IFN‐γ, their DNA methylome does not largely overlap with the one from Th1 cells. Instead, we observed a closer epigenetic similarity between ncTh1 and Th17 cells on a genome‐wide level. Based on our DNA methylome comparisons, we identified marker regions suitable for characterizing these human Th cell lineages. Studies of promoter‐located DMRs in *GSPT1, SRSF7, SLAMF8, SLAMF7, TIGIT*, and *PDCD1 (PD‐1)* by luciferase reporter assays in primary CD4^+^ T cells revealed transcriptional activity that depended on the methylation status. We found an expression of SLAMF7 in stimulated IFN‐γ producing T cells, but not in Tn or IL17A producing T cells. Finally, subsets of SLAMF7^+^ cells were detected among both Th1 and ncTh1 cells. Overall, our findings improve the characterization of human Th cell subsets, enable the identification of functionally relevant molecules, and provide insights into the plasticity of Th cells under inflammatory conditions.

## RESULTS

### Whole‐genome methylome analysis of human Th cell subsets supports close relationship between Th17 and nonclassic Th1 cells

**Figure 1:**
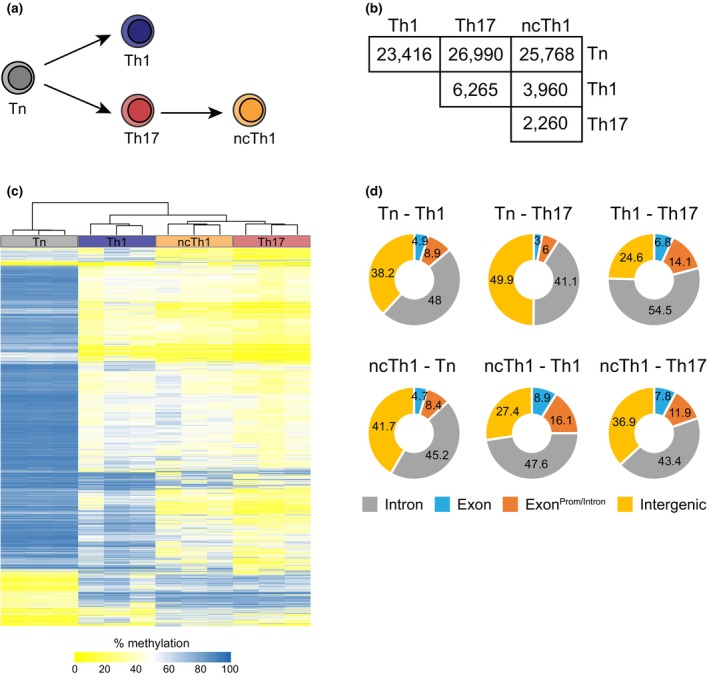
Identification of DMRs by comparing the methylomes of human CD4^+^ T helper cells. After isolation of CD4^+^ Tn cells and effector/memory cells Th1, Th17, and ncTh1 cells **(a)**, their genomic DNA was subjected to WBGS. **(b)** Pairwise comparisons of the methylomes by using *BSmooth* identified DMRs. **(c)** Hierarchical cluster analysis shows the similarities between the samples and sample groups (Tn, Th1, Th17, ncTh1) as well as the clustering of the top 20,000 DMRs based on row variance. Each sample group consisted of cells from three individual donors. **(d)** The plots show the genomic locations of the DMRs: introns, exons, exons with extensions into promoters or introns (Exon^Prom/Intron^), and intergenic regions. Numbers indicate frequencies.

For the proinflammatory CD4^+^ T‐cell subsets Th1, Th17, and ncTh1, which can be associated with chronic inflammatory responses,^25^, ^26^ we have shown that ncTh1 cells develop from Th17 cells as the expression of RORγt and IL‐17A is associated with persistent demethylation of their gene loci (Figure 1a).^18^ However, knowledge on genome‐wide epigenetic changes in DNA methylation upon T‐cell differentiation is restricted to total CD4^+^ memory T cells.^23^ Therefore, we decided to perform genome‐wide DNA methylation analyses in Th1, Th17, and ncTh1 cells to learn more about their similarities and differences in epigenetic regulation of gene expression. First, we isolated genomic DNA from Tn cells, obtained from healthy donors by fluorescence‐activated cell sorting, as well as from Th1, Th17, and ncTh1 cell clones that were selected by cytokine expression (Supplementary figure 1) as recently described.^27^ Samples were subjected to whole‐genome bisulfite sequencing (WGBS), and the generated methylomes were used to identify DMRs by pairwise comparisons using the *BSmooth* software (Supplementary Tables 1–6).^28^ The highest number of DMRs (26,990) was found when comparing Tn cells with Th17 cells, followed by ncTh1 (25 768) and Th1 cells (23 416) (Figure 1b). Although Th1 cells exhibit fewer DMRs in these comparisons, the number of hypomethylated DMRs between Th1 and ncTh1 cells is quite similar (Supplementary figure 2a). However, we identified approximately twice as many hypermethylated regions in ncTh1 cells. Since Th17 cells also exhibit fewer hypermethylated regions, this finding suggests stronger transcriptional restrictions in ncTh1 cells. In pairwise comparisons of antigen‐experienced cells, the highest number of DMRs was observed between Th1 and Th17 cells (6265), followed by the comparison of Th1 with ncTh1 cells (3960) and Th17 with ncTh1 cells (2260). Not unexpectedly, the hierarchical cluster analysis and the number of DMRs indicated a closer relationship between Th17 and ncTh1 cells than with Th1 cells (Figure 1b, c). This hypothesis is supported by a PCA analysis showing a clear separation of Tn and Th1 samples from the clustered sample groups Th17 and ncTh1 (Supplementary figure 2b). Furthermore, gene ontology analyses of hypomethylated DMR‐associated genes obtained from comparisons of Th1 and Th17 methylomes with the ncTh1 methylomes revealed substantial similarities in processes between Th17 and ncTh1 cells, such as protein–protein interactions and positive regulation of biological processes (Supplementary Tables 7–10). By contrast, the analysis of hypomethylated ncTh1 DMRs in the Th17 vs. ncTh1 comparison resulted only in a few pathways linked to development, metabolism, and the immune system. Additionally, the analysis of the ncTh1 hypomethylated regions revealed an overrepresentation of the KEGG pathway “Th1 and Th2 differentiation.” The majority of DMRs were mapped to intronic regions in all pairwise comparisons, followed by intergenic regions (Figure 1d). Interestingly, comparisons between antigen‐experienced T cells reveal a considerable number of DMRs that extend beyond the exons and protrude into promoters and introns (classified as Exon^Prom/Intron^). In summary, the development from a naive CD4^+^ T cell to an antigen‐experienced Th cell subset is accompanied by major changes at the DNA methylation level. This data set supports the ncTh1 cell origin hypothesis, as these cells are more closely related to Th17 cells at the epigenetic level than to Th1 cells.

### The methylome of ncTh1 cells overlaps with both Th1 and Th17 cells to varying degrees

**Figure 2:**
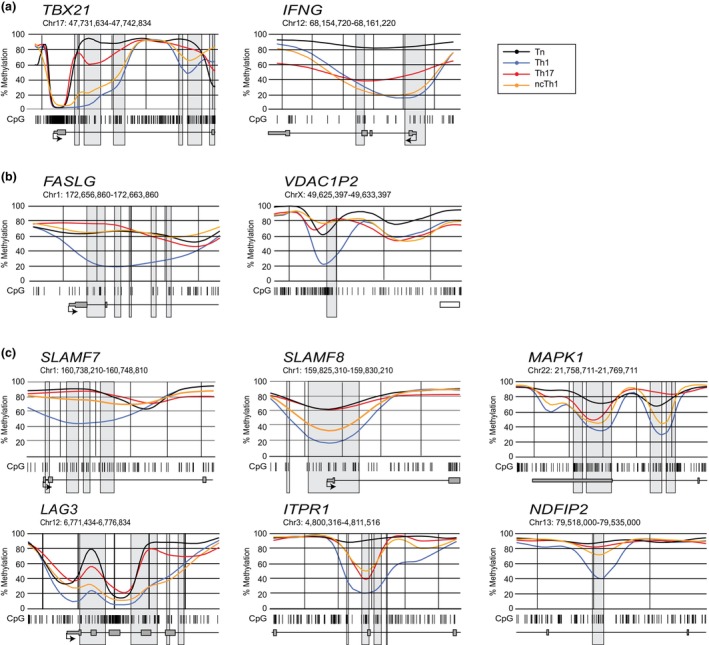
Region plots visualize the DMRs highly demethylated in Th1 cells. Regional methylation curves plotted with *BSmooth* show the course of CpG (barcode) mean methylation of Tn (black), Th1 (blue), Th17 (red), and ncTh1 (orange) cells at the indicated chromosomal location and gene. DMRs identified in pairwise comparisons with Tn cells are shown as gray boxes. **(a)** Region plots of Th1 cell lineage genes *TBX21* and *IFNG*, **(b)** *FASLG* and *VDAC1P2* containing uniquely demethylated regions, and **(c)** gene loci showing similar methylation patterns in Th17 and ncTh1 cells. Plots show the smoothed, mean methylation values obtained from three independent samples in each group.

The lower number of DMRs between Th17 and ncTh1 cells and the close relationship revealed by the PCA analysis is in accordance with our recent finding on the development of ncTh1 cells from Th17 cells or their precursors.^18^ Although the hierarchical cluster analysis of ncTh1 and Th17 cells produced similar patterns (Figure 1c), we identified a marked number of regions showing differential methylation. This raises the question of whether these differences occur in the same gene loci to different extents, or in completely different genes. Line plots showing the levels of gene locus methylation confirmed the expected demethylation of the first *TBX21* intron in Th1 and ncTh1 cells and the methylation status in Tn or Th17 cells. Similar observations were made for *IFNG* (Figure 2a). In contrast, the highly demethylated regions in *FASLG* and *VDAC1P2* were found to be unique to Th1 cells (Figure 2b). Interestingly, we observed a similar curve shape for several genes with a more pronounced demethylation in Th1 cells than in ncTh1 cells, but at a higher methylation level (Figure 2c). High, medium, and low differences in curve shape were observed in the following gene loci: *SLAMF7*, *SLAMF8*, *MAPK1*, *LAG3*, *ITPR1*, and *NDFIP2*.

**Figure 3:**
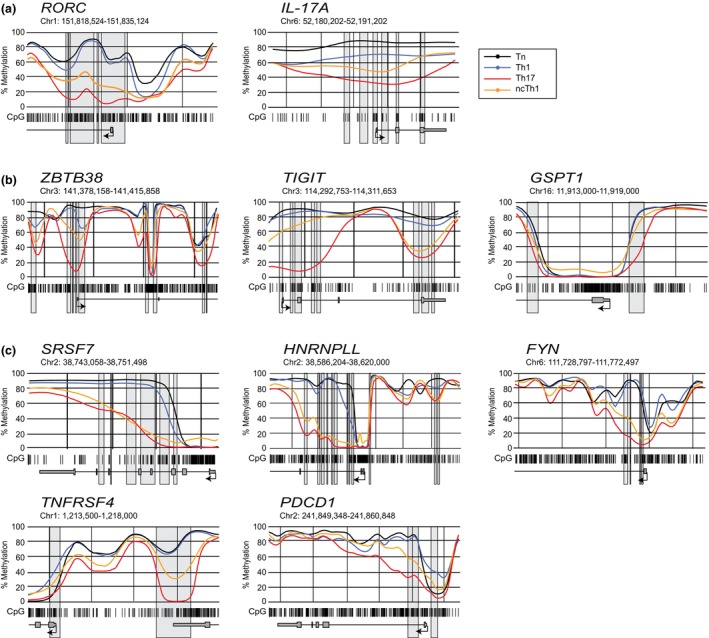
Region plots visualize the DMRs highly demethylated in Th17 cells. Regional methylation curves plotted with *BSmooth* show the course of CpG (barcode) mean methylation of Tn (black), Th1 (blue), Th17 (red), and ncTh1 (orange) cells at the indicated chromosomal location and gene. DMRs identified in pairwise comparisons with Tn cells are shown as gray boxes. **(a)** Region plots of Th17 cell lineage genes *RORC* and *IL17A*, **(b)** *ZBTB38*, *TIGIT*, and *GSPT1* containing uniquely demethylated regions, and **(c)** gene loci showing similar methylation patterns in Th17 and ncTh1 cells. Plots show the smoothed, mean methylation values obtained from three independent samples in each group.

**Figure 4:**
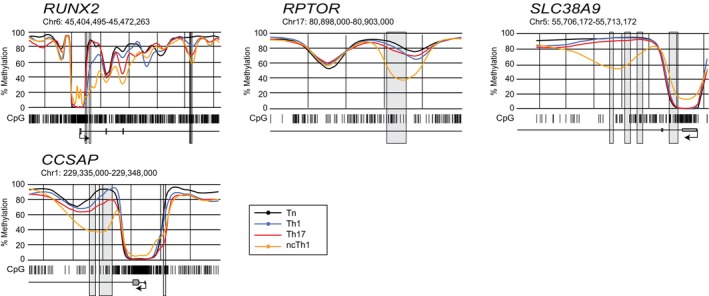
Region plots visualize the DMRs highly demethylated in ncTh1 cells. Regional methylation curves plotted with *BSmooth* show the course of CpG (barcode) mean methylation of Tn (black), Th1 (blue), Th17 (red), and ncTh1 (orange) cells at the indicated chromosomal location and gene. DMRs identified in pairwise comparisons with Tn cells are shown as gray boxes. Region plots of gene loci containing highly demethylated regions in ncTh1 cells are depicted. Plots show the smoothed mean methylation values obtained from three independent samples in each group.

The methylation level in *RORC* was lower in Th17 and ncTh1 cells compared with Tn and Th1 cells, but surprisingly to varying degrees (Figure 3a). Similarly, less pronounced demethylation was found in ncTh1 for *IL17A*, presumably due to the epigenetic regulation of RORC. Partially overlapping methylation levels between Th17 and ncTh1 cells were identified in *ZBTB38*, *TIGIT*, and *GSPT1* (Figure 3b). We observed similar curve shapes between Th17 and ncTh1 cells for *SRSF7*, *HNRNPLL*, and *FYN* (Figure 3c), whereas increased methylation levels were found in *TNFRSF4* and *PDCD1* of ncTh1 cells. Highly demethylated regions in the ncTh1 cell methylome were identified in the transcription factor gene *RUNX2*, the growth regulator gene *RPTOR*, the transporter gene *SLC38A9*, and the microtubule stabilizer gene *CCSAP* (Figure 4). Similar to *TBX21*, the promoters of *RUNX2*, *SLC38A9*, and *CCSAP* are demethylated in all CD4^+^ T‐cell populations, but the intronic regions are more demethylated in ncTh1 cells. Thus, the degree of overlap in the methylome between ncTh1 and Th1 or Th17 varies, suggesting an incomplete copy of Th1‐ or Th17‐specific epigenetic programs.

### Several Th cell subset‐specific DMRs display a methylation‐dependent transcriptional activity

**Figure 5:**
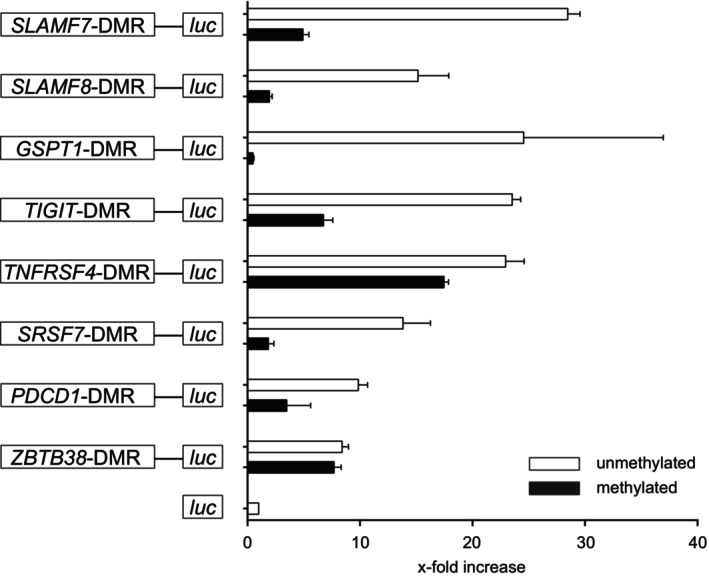
Several DMRs display transcriptional activity in a methylation‐dependent manner. DMRs from the indicated gene loci were cloned into a firefly luciferase reporter plasmid pCpGL‐mcs‐basic and transfected into stimulated primary CD4^+^ T cells either unmethylated (white bars) or methylated (black bars) together with a pRL‐TK Renilla control plasmid. After 24 h of cultivation, cells were harvested and subjected to a dual‐luciferase reporter assay. Firefly light units were normalized to Renilla units, and the x‐fold value was calculated by using a construct without DMR (*luc*). The graph shows the mean of triplicates obtained from one out of two to three independent experiments.

Upon TCR activation, CD4^+^ Tn cells can differentiate into specific Th cell lineages, depending on the signal transduction events that are triggered by mediators and provided by antigen‐presenting cells.^29^ Initial events lead to the activation/upregulation of factors that initiate lineage‐specific transcriptional programs. Regulatory events, such as specific methylation or demethylation, help to maintain specific transcriptional patterns and thus the phenotype of a lineage. To answer the question of whether the identified DMRs can mediate transcriptional activity as promoters, and whether the regulation depends on the methylation status, we cloned selected DMRs into CpG‐free luciferase reporter plasmids, which we then used in dual‐luciferase reporter assays.^30^ Transfection of the reporter constructs into primary human CD4^+^ T cells revealed an 8‐ to 30‐fold transcriptional activity mediated by DMRs located in the promoter region of *SLAMF7*, *SLAMF8, GSPT1*, *TIGIT*, *TNFRSF4*, *SRSF7*, *PDCD1*, and *ZBTB38* (Figure 5). The CpG‐free luciferase reporter plasmid allowed artificial methylation of all CpG motifs within the DMRs introduced by cloning. Methylation of the cloned DMRs dramatically reduced the reporter activity in all constructs except for those in which we cloned regions of *TNFRSF4* and *ZBTB38*. Based on these reporter assays, we conclude that DMRs located in *GSPT1*, *SRSF7*, *SLAMF8*, *SLAMF7*, *TIGIT*, and *PDCD1* act as promoters and can mediate transcriptional activity in a methylation‐dependent manner.

### SLAMF7 is exclusively expressed in IFN‐γ producing Th cell subsets

**Figure 6:**
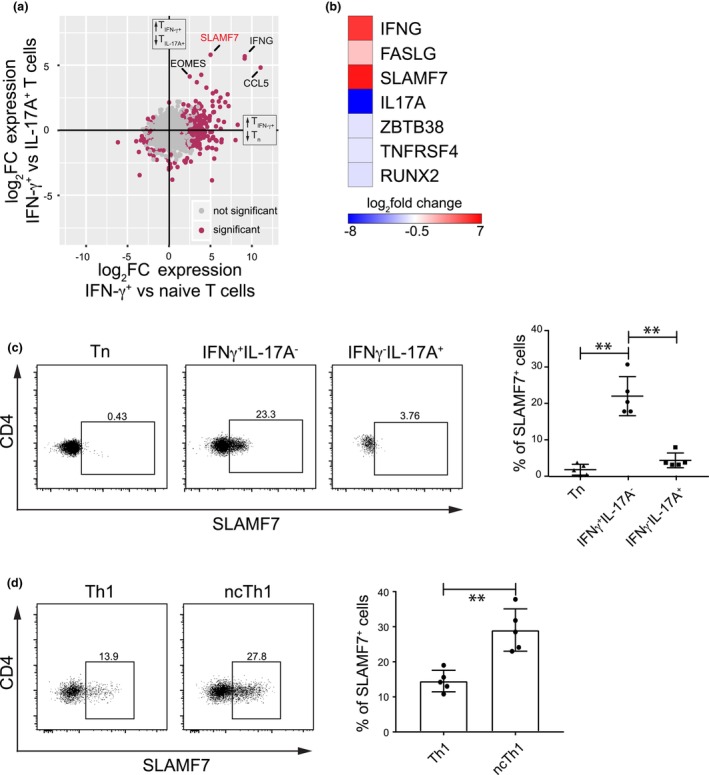
SLAMF7 is expressed in IFN‐γ producing cells. Human primary CD4^+^ T cells were stimulated with PMA and ionomycin for 2 h, labeled by using a cytokine secretion assay for IFN‐γ and IL‐17A, sorted into Tn cells, IFN‐γ producing or IL‐17A producing memory T cells, and subjected to RNA‐seq. **(a)** Pairwise comparisons identified genes uniquely expressed in IFN‐γ producing cells. Up‐ and downregulation of genes in the analyzed T‐cell populations is indicated by arrows. **(b)** DMR‐associated genes show differential expression when comparing IFN‐γ or IL‐17A producing memory T cells. Log_2_fold change values from Supplementary Table 13 were multiplied by −1 and converted into a blue‐white‐red color code. **(c)** Human primary CD4^+^ T cells were stimulated by anti‐CD3/CD28 antibodies for 4 h, labeled by using fluorescence‐conjugated antibodies and analyzed by flow cytometry. SLAMF7 expression was analyzed in Tn cells, IFN‐γ^+^ or IL‐17A^+^ memory T cells, pregated on IFN‐γ^−^IL‐17A^−^CD45RA^+^, IFN‐γ^+^IL‐17A^−^ or IFN‐γ^−^IL‐17A^+^ cells, respectively. The summary plot on the right shows the frequency of CD4^+^SLAMF7^+^ cells from five independent experiments. **(d)** SLAMF7 expression was measured in stimulated CD4+ T cells (see 6c) using flow cytometry by pregating on Th1 (IFN‐γ^+^CD161^−^) or Th17 (IFN‐γ^+^CD161^+^) cells. The summary plot on the right shows the frequency of CD4^+^SLAMF7^+^ cells from five independent experiments. *P < 0.05; **P < 0.01; ***P < 0.001; ****P < 0.0001; Wilcoxon signed‐rank test.

Since epigenetic regulation at the DNA level can act as a transcriptional on/off switch or allow a faster and stronger response to environmental changes, we were interested in identifying differentially expressed genes (DEGs) in IFN‐γ producing cells, including both Th1 and ncTh1 cells. Therefore, we stimulated human primary CD4^+^ T cells with PMA and ionomycin for 2 h, labeled them using a cytokine secretion assay for IFN‐γ and IL‐17A, and sorted them into Tn, IFN‐γ secreting, or IL‐17A secreting memory T cells. We then subjected them to RNA‐seq and analyzed them using pairwise comparisons (Supplementary Tables 11–13). The short‐term stimulated memory T cells showed a substantial and unique upregulation of *SLAMF7* and *IFNG* expression (Figure 6a), both associated with DMRs, in IFN‐γ expressing cells. Interestingly, among the top DEGs, we identified *EOMES*, a transcription factor that is involved in ncTh1 cell development.^19^ Focusing on the DMRs described in Figures 2, 3, 4, we detected substantial expression differences between Th1 and Th17 cells in the DMR‐associated gene loci of *IFNG*, *FASLG*, *SLAMF7*, *IL17A*, *ZBTB38*, *TNFRSF4*, and *RUNX2* (Figure 6b). Considering that SLAMF7 is a target in cancer therapy,^31^ its epigenetic regulation in Th1 cells and differential expression in IFN‐γ producing memory CD4^+^ T cells encouraged us to take a closer look at the SLAMF7 expression at the protein level in TCR‐triggered CD4^+^ T cells. In accordance with the RNA‐seq data, we observed SLAMF7 expression in IFN‐γ^+^IL‐17A^−^ cells, but not in IFN‐γ^−^IL‐17A^+^ or Tn cells (Figure 6c). By further gating the IFN‐γ^+^IL‐17A^−^ population into Th1 and ncTh1 with the help of CD161, we detected SLAMF7^+^ cells in both populations, yet a significantly higher proportion of positive cells was found in ncTh1 cells (Figure 6d). In conclusion, we identified SLAMF7 expression exclusively in IFN‐γ producing T cells, with a particularly high frequency of positive cells among ncTh1 cells.

## DISCUSSION

The aim of this study was to gain insight into the epigenetic regulation of gene expression in proinflammatory CD4^+^ memory T cells. Therefore, Tn cells, which represent the starting point for memory T‐cell development, and well‐characterized Th1, Th17, and ncTh1 T‐cell clones were subjected to a genome‐wide methylation analysis. The reorganization of gene expression toward a memory T‐cell population was clearly visible by the large number of identified DMRs in Th1, Th17, and ncTh1 cells when compared with Tn cells. The close clustering of Th17 and ncTh1 samples, along with the overlaps in the gene ontology analyses, indicates a partial overlap of transcriptional regulatory networks and confirms our previous data that ncTh1 cells develop from Th17 cells.^18^ The well‐studied epigenetically regulated transcription factor genes *TBX21* and *RORC*, as well as the corresponding cytokine genes *IFNG* and *IL17A*, showed a lineage‐specific differential methylation in Th1 and Th17 cells, but not in ncTh1 cells, in which we observed overlapping patterns for all four gene loci. In addition, we found DMRs that were exclusively demethylated in ncTh1 cells, such as the transcription factor encoding gene *RUNX2*. A detailed analysis of selected DMRs by luciferase reporter assays consistently showed transcriptional activity that was methylation‐dependent in most cases. Short‐term stimulation experiments followed by RNA‐seq analysis revealed transcription of the DMR‐containing genes *IFNG*, *FASLG*, *SLAMF7*, *IL‐17A*, *ZBTB38*, *TNFRSF4*, and *RUNX2*, suggesting that the DMRs contribute to their expression under these conditions. Finally, we focused on the SLAMF7 expression in stimulated T cells. We could not identify any protein expression in Tn and Th17 cells, but we did observe expression in Th1 and particularly ncTh1 cells.

The identification of the DMRs from the pairwise comparisons of the methylomes of Tn, Th1, Th17, and ncTh1 cells clearly reflects the differentiation‐dependent effects on methylome changes as described previously for central memory (Tcm), effector memory (Tem), and CD45RA^+^ memory T cells.^23^ In our lineage‐specific approach, we also detected known memory‐associated genes that are epigenetically regulated, such as *HNRNPLL*, *LAG3*, *PDCD1*, or *TBX21*. Interestingly, we identified RUNX2 expression in IFN‐γ‐secreting cells and a comparable higher demethylation in the first intron of its gene locus in ncTh1 cells. Since RUNX2 binding sites were found in open chromatin regions by pairwise comparison of human Tcm and Tem, and the transcriptional Th1 cell program repressor Gfi1 was shown to target the key genes *Tbx21*, *Eomes*, and *Runx2* in mice, we hypothesize that this transcription factor is important for ncTh1 cell function.^23^, ^32^

Juvenile idiopathic arthritis (JIA) is a chronic inflammation of the joints that begins in childhood.^33^ Our recent work suggests that a combination of proinflammatory Th cell subsets drives the inflammation in the joints, starting with infiltrating Th1 and Th17 cells.^26^ However, the cytokine milieu in the joints, particularly IL‐12, pushes the Th17 cell transcriptome toward type 1 immunity, which results in Th17/Th1 cells expressing both IFN‐γ and IL‐17A. Further development leads to ncTh1 cells that stop producing IL‐17A. Remarkably, both our previous candidate approach and the genome‐wide analysis in the present study indicate an increase in methylation in epigenetically regulated Th17 genes, but not a complete shut‐off scenario compared with Tn cells. Thus, Th17 cells seem to be able to adapt in certain inflammatory environments, but the epigenetic control at the DNA methylation level does not completely switch to a Th1 cell methylome. Since our DNA methylation analysis, although limited to characterized T‐cell clones, is based on pooled genomes, we cannot completely exclude that a certain percentage of cells show a complete switch, while others maintain their original pattern. In this case, the DNA methylation pattern could be interpreted as a mixture of intermediate and terminal states.

Another interesting aspect of Th cell flexibility is the development into pathogenic GM‐CSF producing cells upon triggering of IL1‐R and IL‐23R.^34^, ^35^ GM‐CSF expression was detected in inflamed joints and positively correlated with EOMES expression in the synovial fluid of JIA patients,^19^, ^36^ leading to the hypothesis that there is a common regulatory pathway. Our present study confirms EOMES expression in both IFN‐γ and IL‐17A‐producing cells but did not detect a DMR in the transcription factor gene locus in Th17 or ncTh1 cells compared with Tn cells. An elegant study using a fate reporter approach identified a significant enrichment of RUNX2 accessible regions in GM‐CSF expressing Th cells.^35^ As our study identified a DMR in the first intron of *RUNX2* in ncTh1 and Th1 cells, it is tempting to speculate that pathogenic Th cells are controlled by both EOMES and RUNX2. Further single‐cell transcriptomic and chromatin accessibility assays are needed to clarify the regulatory networks in pathogenic T cells and their lineage identity.

SLAMF7 is a transmembrane receptor that mediates lymphocyte activation and is expressed on various immune cell populations, such as NK cells, CD4^+^ and CD8^+^ T cells, and B cells. The high expression of SLAMF7 in multiple myeloma made it an attractive therapeutic target. One strategy is to use SLAMF7‐binding antibodies like Elotuzumab,^37^ while another strategy is to target multiple myeloma directly using CAR T cells against SLAMF7.^38^ Our DNA methylation analysis revealed an epigenetic regulation of the *SLAMF7* gene locus in Th1 and ncTh1 cells, with only a small difference in the latter compared with Tn cells. RNA‐seq and protein expression data support the findings of the DNA methylation analysis by a selected expression of SLAMF7 in IFN‐γ producing cells, but not in Tn or IL‐17A producing cells. The incompletely demethylated region in *SLAMF7* suggested the presence of a subpopulation of epigenetically regulated cells, which was confirmed by the expression in subsets of Th1 and ncTh1 cells. A recent study on T cells defined SLAMF7^+^ cells as the cytotoxic arm of the T‐cell pool.^39^ Therefore, it is of great interest to clarify in further studies whether the SLAMF7^+^CD4^+^ T cells are the main driver of tissue destruction in chronic inflammation that can be eliminated using therapeutic antibodies or CAR T cells.

## METHODS

### Human donors

Human samples were obtained from the Institute of Transfusion Medicine and Transplant Engineering, Hannover Medical School (MHH). Written informed consent was obtained from all donors (MHH Ethics Committee vote 3639–2017). T helper cell clones (Th1, Th17, ncTh1 cell clones) were generated as recently described.^27^ The procedures were in accordance with the ethical standards of the Regional Committee for Human Experimentation of the University of Florence, Italy (No. 2016‐246‐OSS 105/12).

### Antibodies and flow cytometry

All monoclonal antibodies used for sorting and immunophenotyping were purchased from Biolegend and BD Biosciences. The Foxp3/Transcription Factor staining buffer set (Thermo Fisher Scientific) was used for intracellular staining. In this study, cells were labeled with Alexa‐Fluor 488‐conjugated anti‐human CD3 (clone UCHT1), BV605‐conjugated anti‐human CD3 (clone UCHT1), PE‐Cy7‐conjugated anti‐human CD4 (RPA‐T4), PerCP/Cy5‐conjugated anti‐human CD45RA (clone HI100), BV421‐conjugated anti‐human CD127 (clone HIL‐7R‐M21), APC‐conjugated anti‐human CD25 (clone BC96), PE‐conjugated anti‐human CD161 (clone DX12), Alexa‐Fluor 647‐conjugated anti‐human IL17α (clone BL168), and V500‐conjugated anti‐human IFNγ (clone B27). The Foxp3/Transcription Factor staining buffer set (Thermo Fisher Scientific) was used for intracellular staining of PMA (10 ng/mL; Sigma Aldrich) and ionomycin (0.5 μg/mL; Sigma Aldrich) stimulated CD4^+^ T cells (Brefeldin A (Sigma Aldrich) added after 2 h; 10 μg/mL; 4‐h total stimulation time). Cells were sorted by FACS Aria‐II SORP or FACS Aria‐IIu (both BD Biosciences) and analyzed by LSR Fortessa (BD Biosciences). Flow cytometric data were analyzed using FlowJo software (BD Biosciences).

### Human sampling and cell isolation

For WGBS, the Th1, Th17, and ncTh1 cell samples were prepared from *ex vivo* isolated, cultured clones as previously described.^27^ Tn cells for WGBS, as well as cells for luciferase reporter assays, RNA‐seq, or phenotypic characterization were obtained from PBMCs isolated from leukocyte reduction system (LRS) cones using Ficoll‐based density gradient centrifugation (Lymphoprep; StemCell Technologies) and SepMate tubes (StemCell Technologies). Total CD4^+^ T cells were purified using the RosetteSep human CD4^+^ T‐cell enrichment kit (StemCell Technologies) and sorted into Tn cells (CD3^+^CD4^+^CD45RA^+^), subjected to luciferase reporter assays, or further separated into CD4^+^CD45RA^+^ and CD4^+^CD45RA^−^ cells by using anti‐CD45RA MicroBeads (Miltenyi Biotec) and the autoMACS Pro magnetic separation system (Miltenyi Biotec). CD4^+^CD45RA^+^ and CD4^+^CD45RA^−^ cells were stimulated with PMA (10 ng/mL; Sigma Aldrich) and ionomycin (0.5 μg/mL; Sigma Aldrich) for 2 h and sorted by using the IFN‐γ (FITC) and IL17A (PE) cytokine secretion assays according to the manufacturer's protocol (Miltenyi Biotec). Cytokine‐positive and ‐negative populations were labeled with fluorescence‐conjugated antibodies and sorted into naive T cells (CD4^+^CD45RA^+^CD3^+^CD127^high^CD25^−^IFN‐γ^−^IL17A^−^), Th1 cells (CD4^+^CD45RA^−^CD3^+^CD127^high^CD25^−^IFN‐γ^+^IL17A^−^), and Th17 cells (CD4^+^CD45RA^−^CD3^+^CD127^high^CD25^−^IFN‐γ^−^IL17A^+^) by flow cytometry. Human regulatory T cells (CD4^+^CD3^+^CD127^low^CD25^+^) were excluded from the sorting.

### Whole‐genome bisulfite sequencing

Genomic DNA (gDNA) was isolated from *ex vivo* isolated Tn cells and Th1, Th17 and ncTh1 cell clones by using the DNeasy Blood & Tissue Kit (Qiagen) according to the manufacturer's instructions. Fragmentation of gDNA was performed using a Covaris S2 (Covaris), at 10% duty cycle, intensity 4 and 200 cycles per burst for 80 s, to obtain fragments with an average length of 300 bp. The size of the fragments was verified using an Agilent Technologies 2100 Bioanalyzer. DNA sequencing libraries were prepared from the fragmented gDNA using the TruSeq DNA Sample Prep Kit v2 (Illumina) according to the manufacturer's instructions, adding an extra step to the workflow: after ligation of the adapter molecules to the DNA fragments, the sample was subjected to bisulfite conversion reaction using the EZ DNA Methylation Kit (Zymo Research). The bisulfite converted library was amplified by PCR using the TruSeq primer mix and the KAPA Hifi Uracil/Polymerase Master Mix (Kapa Biosystems). The PCR product was purified and size controlled by using a 2100 Bioanalyzer (Agilent Technologies). Libraries were sequenced on an Illumina HiSeq2500 sequencer using the TruSeq SBS Kit v3‐HS (Illumina, 200 cycles, paired‐end sequencing) with an average of 2 × 10^8^ reads per replicate.

The sequenced 2 × 100 bp paired‐end libraries were checked for sufficient sequencing quality and potential adapter contamination using the programs *FastQC* (Babraham Bioinformatics, https://www.bioinformatics.babraham.ac.uk/projects/fastqc/), *trim_galore* (version 0.6.5; Babraham Bioinformatics, https://www.bioinformatics.ac.uk/projects/trim_galore/) and *cutadapt*.^40^ Quality‐controlled libraries were mapped against the human reference genome (GRCh38) using the bisulfite short read mapping software *BSMAP*.^41^ Only unique, properly paired reads (*methratio.py* parameters: ‐‐unique, ‐‐paired, ‐‐remove‐duplicate) were used to determine CpG methylation levels and coverage. CpG motifs with a minimum coverage of five mapped reads in at least two replicates of a condition served as input for methylation level smoothing and DMR detection using the Bioconductor package *bsseq* (version 1.16).^28^ Regions were classified as differentially methylated between two conditions if (1) they contain at least three CpG motifs with (2) a maximum distance of 200 bases, (3) a mean methylation difference of at least 0.25, and (4) all CpGs in the region have an associated t‐statistic (computed with the *bsseq* function *Bsmooth.tstat*) within the 0.01 to 0.99 quantile interval (parameter qcutoff of the *bsseq* function *dmrFinder*). The resulting DMRs were further classified according to their genomic location using human annotations from Ensembl.^42^

Hierarchical clustering and principal component analysis of DMR data were performed as recently described.^43^, ^44^ GO analysis with the DMR‐associated genes from the pairwise comparisons of CD4^+^ memory T cells was conducted by using the g:Profiler tool.^45^ This GO analysis included terms relating to molecular functions, biological processes, cellular components, and the Kyoto Encyclopedia of Genes and Genomes (KEGG) pathways.

### Plasmids and luciferase reporter assays

The CpG‐free firefly luciferase reporter vector pCpGL‐mcs‐basic was generated by inserting the oligonucleotide mcs (Supplementary Table 14) into the vector pCpGL‐basic by using the restriction sites *PstI* and *HindIII*.^30^ Selected DMRs were amplified by PCR using specific primer pairs (Supplementary Table 14) and cloned into pCpGL‐mcs‐basic by using the restriction enzymes *BamHI* and *HindIII* or *SpeI*. *In vitro* methylation of the generated constructs was performed using the *M.SssI* CpG methyltransferase (New England Biolabs) according to the manufacturer's instructions. Complete methylation was controlled by incubation with the methyl‐sensitive restriction enzyme *HpaII*. Primary human CD4^+^ T cells were stimulated with plate‐bound anti‐human CD3/CD28 antibodies (1 μg/mL each) for 72 h. One million cells were transfected with the verified demethylated or methylated DMR reporter plasmids together with the pRL‐TK plasmid encoding the Renilla luciferase by using the Human T Cell Nucleofector Kit (Lonza) according to the manufacturer's protocol. After 4 h of cultivation, the cells were restimulated with phorbol 12‐myristate 13‐acetate (PMA; 1 ng/mL; Sigma Aldrich) and ionomycin (0.5 μg/mL; Sigma Aldrich) in the presence of 1000 U/mL Human IL‐2 IS (Miltenyi Biotec). Cells were harvested 24 h after restimulation, lysed, and subjected to a dual‐luciferase reporter assay (Promega) according to the manufacturer's instructions. Firefly light units were normalized to the Renilla light units, and x‐fold differences were calculated by using a plasmid without DMR.

### RNA‐seq

Total RNA was extracted from FACS‐sorted stimulated human Tn, Th1, and Th17 cells using the RNeasy Micro Kit (Qiagen). cDNA was synthesized and amplified using template switching technology of the SMART‐Seq v4 Ultra Low Input RNA Kit (Clontech Laboratories), followed by purification using the Agencourt AMPure XP Kit (Beckman Coulter). Library preparation was performed with Nextera XT DNA Library Prep Kit (Illumina). Deep sequencing was carried out on Illumina HiSeq2500 using 100 bp paired‐end reads. Sequenced reads were trimmed using Cutadapt^29^ and then aligned to human reference genome GRCh38 using STAR.^46^ Next, gene‐level counts were calculated using tximport^47^ from transcript counts and served as input to DESeq2^48^ for pairwise detection and quantification of differential gene expression. Genes with an adjusted *P*‐value of less than 0.05 were considered as statistically significant.

### Statistical analysis

For WGBS and RNA‐seq analysis the statistical values were calculated using the *bsseq* and *DESeq2* software packages as described above. Statistical analysis of the SLAMF7 expression was performed using the GraphPad Prism software. Here, the Wilcoxon signed‐rank test was applied to compare the samples between the two conditions. All data are presented as mean or mean ± SD, and *P*‐values <0.05 are considered significant (*P < 0.05; **P < 0.01; ***P < 0.001; ****P < 0.0001; ns, not significant).

## AUTHOR CONTRIBUTIONS

**Anna Ntalli:** Investigation; formal analysis; writing – original draft. **Michael Beckstette:** Methodology; software; data curation. **Saumya Kumar:** Software; formal analysis; data curation. **Laura Maggi:** Resources; writing – original draft. **Francesco Annunziato:** Resources; writing – review and editing. **Luis Graca:** Resources; writing – review and editing. **Stefan Floess:** Conceptualization; data curation; visualization; writing – original draft; writing – review and editing. **Jochen Huehn:** Conceptualization; supervision; project administration; funding acquisition; writing – original draft; writing – review and editing.

## CONFLICT OF INTEREST

The authors have no conflicts of interest to declare.

## Supporting information


Supplementary figure 1.

Supplementary figure 2.

Tables S1–S14.

## Data Availability

Fastq files from WGBS and RNA‐seq used for analysis described in this manuscript have been uploaded to the GEO data repository. The GEO accession codes are GSE292305 and GSE122030.
