# Supplementary material for: DNA methylation profiling of human CD4 + T helper cells reveals the epigenetic control of SLAMF7 expression in IFN‐γ producing cells
Source: Immunol Cell Biol. 2025 Nov 4;104(1):7–19. doi: 10.1111/imcb.70063 (PMC12800728; doi:10.1111/imcb.70063)
Supplement: Supplementary file 1 — Supplementary figure 1. [file IMCB-104-7-s003.pdf]

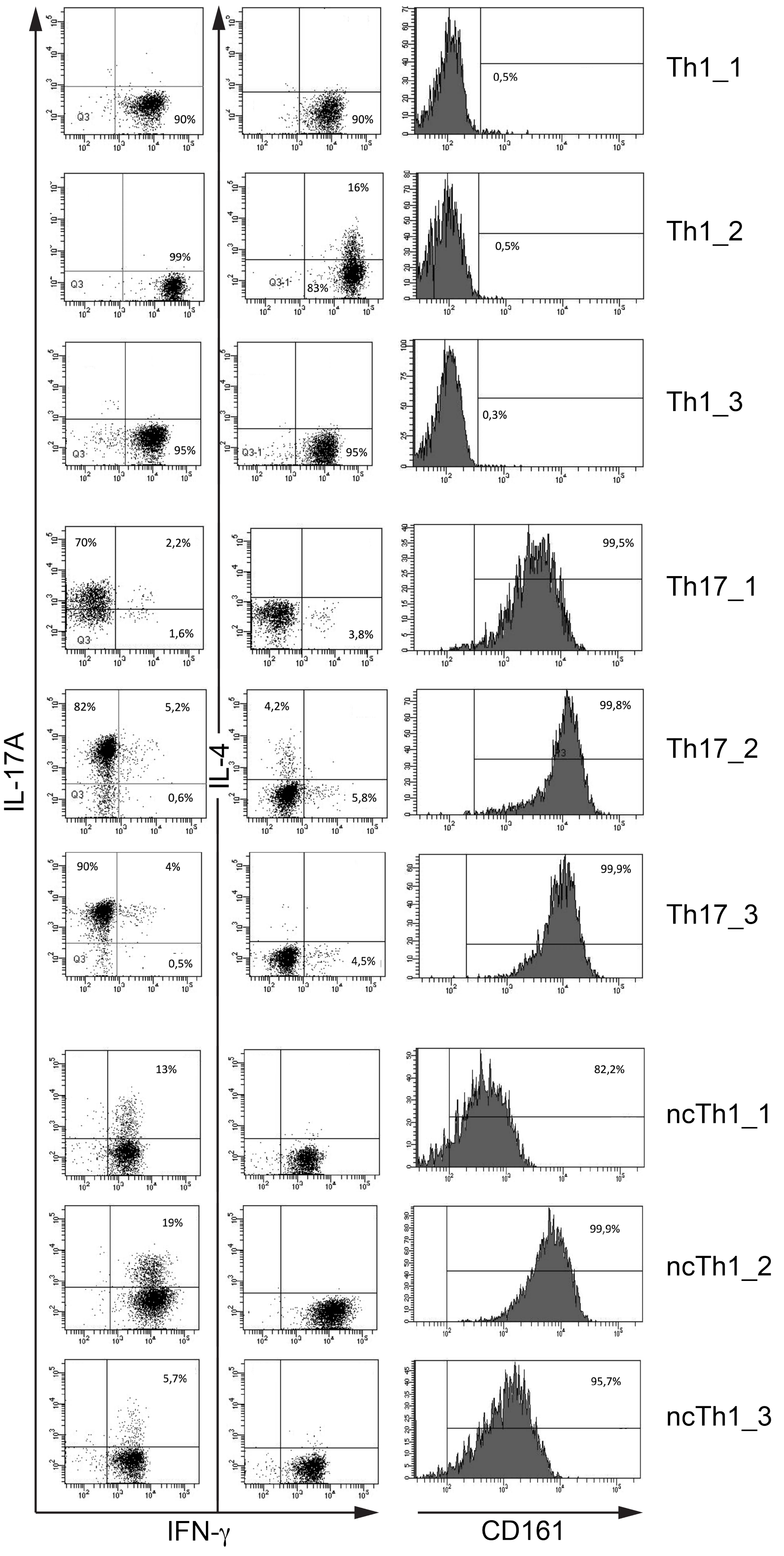

Supplementary Figure 1: Th cell marker expression in Th1, Th17, ncTh1 cell clones. Expression of IL-17A, IL-4, IFN-γ and CD161 was measured by flow cytometry in the indicated T cell clones.
