# Supplementary material for: DNA methylation profiling of human CD4 + T helper cells reveals the epigenetic control of SLAMF7 expression in IFN‐γ producing cells
Source: Immunol Cell Biol. 2025 Nov 4;104(1):7–19. doi: 10.1111/imcb.70063 (PMC12800728; doi:10.1111/imcb.70063)
Supplement: Supplementary file 2 — Supplementary figure 2. [file IMCB-104-7-s001.pdf]

(a)

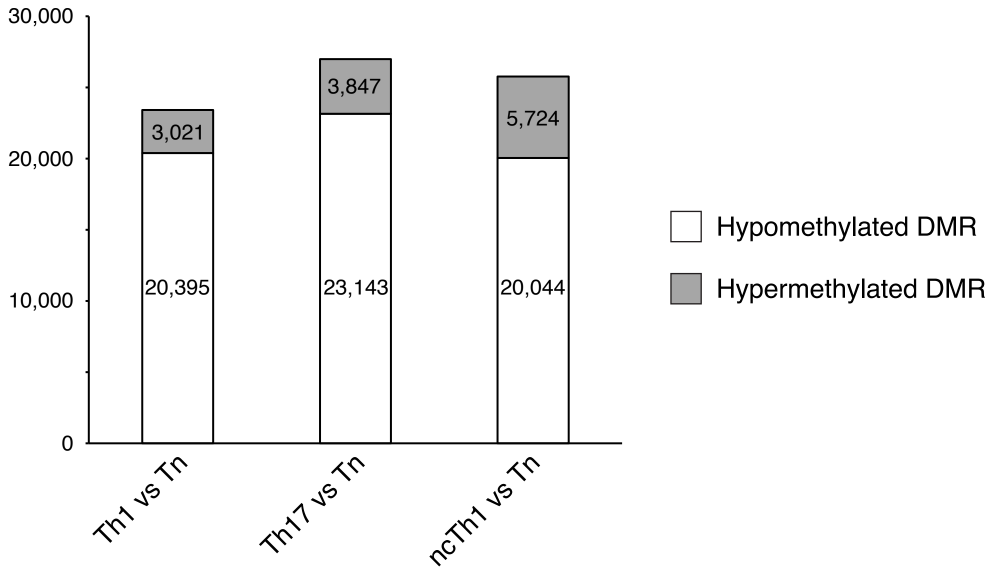

(b)

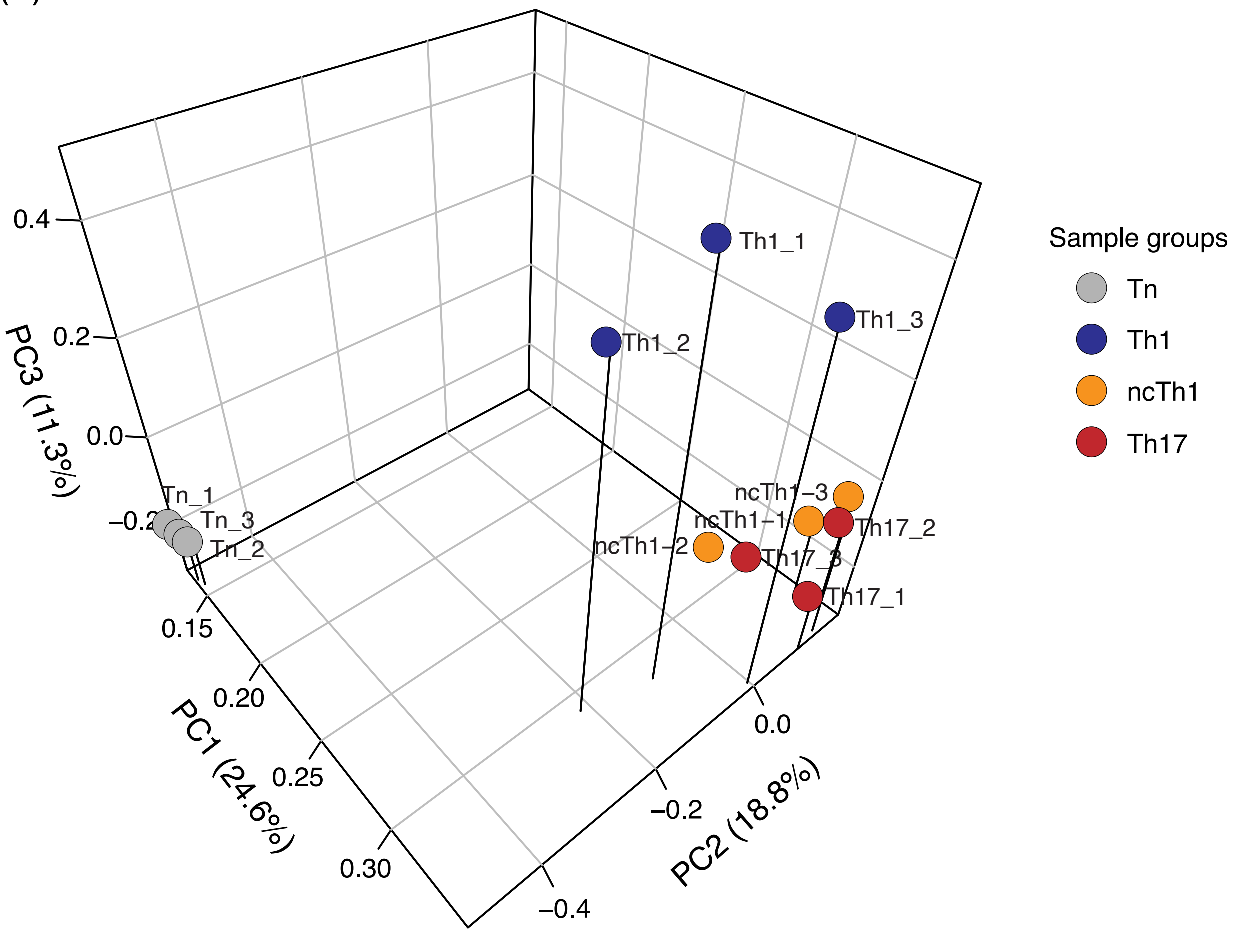

Supplementary Figure 2: Analysis of methylome data generated from human CD4⁺ T helper cell populations. (a) Distribution of DMRs obtained from the Th1 vs Tn, Th17 vs Tn and ncTh1 vs Tn pairwise comparisons in hypo- (clear) and hypermethylated (grey) regions. The number of regions is indicated. (b) Principal component analysis (PCA) of DNA methylation in Tn, Th1, Th17, and ncTh1 cells. PCA was conducted using scaled and centered DMR mean methylation values of the top 50,000 DMRs (based on row variance). Plot shows the principal components 1-3, including the percentage of explained variance. Analysis was performed with three independent replicates per group (color-coded).
